# Fertility preservation practices and gastrointestinal oncologist in Europe: a pan-European study

**DOI:** 10.1093/oncolo/oyaf350

**Published:** 2025-11-05

**Authors:** Irit Ben-Aharon, Tal Goshen-Lago, Alberto Puccini, Maria Alsina, Dirk Arnold, Hanneke van Laarhoven, Anneli Elme, Tineke Buffart, Nina Fokter-Dovnik, Tomas Sokop, Radka Obermanova, Florian Lordick, Demetris Papamichael, Francesco Sclafani, Elena Elez, Julien Taieb

**Affiliations:** Division of Oncology, Rambam Health Care Campus, Haifa 31096, Israel; Rappaport Faculty of Medicine, Technion – Israel Institute of Technology, Haifa 3200003, Israel; Division of Oncology, Rambam Health Care Campus, Haifa 31096, Israel; Department of Biomedical Sciences, Humanitas University, Pieve Emanuele, Milan 20090, Italy; IRCCS Humanitas Research Hospital, Medical Oncology and Hematology Unit, Humanitas Cancer Center, Rozzano, Milan 20089, Italy; Medical Oncology Department, Hospital Universitario de Navarra, Pamplona 31008, Spain; Asklepios Tumor Center Hamburg and AK Altona, Hamburg 22763, Germany; Department of Medical Oncology, Cancer Center Amsterdam, Amsterdam University Medical Center, Amsterdam 1081 HV, The Netherlands; Chemotherapy Center, North Estonia Medical Center Foundation, Tallinn 13419, Estonia; Department of Medical Oncology, Cancer Center Amsterdam, Amsterdam University Medical Center, Amsterdam 1081 HV, The Netherlands; Department of Oncology, University Medical Centre Maribor, Maribor 2000, Slovenia; Department of Comprehensive Cancer Care, Faculty of Medicine, Masaryk University, Brno 625 00, Czech Republic; Department of Comprehensive Cancer Care, Faculty of Medicine, Masaryk University, Brno 625 00, Czech Republic; Department of Medicine II, University Cancer Center Leipzig, Cancer Center Central Germany, University of Leipzig Medical Center, Leipzig 04103, Germany; Department of Medical Oncology, Bank of Cyprus Oncology Center, Nicosia 2029, Cyprus; Department of Gastrointestinal Oncology, Université libre de Bruxelles (ULB), Hôpital Universitaire de Bruxelles (HUB), Institut Jules Bordet, Brussels 1070, Belgium; Medical Oncology Department, Vall d’Hebron University Hospital and Institute of Oncology (VHIO), Universitat Autònoma de Barcelona, Barcelona 08035, Spain; Université Paris Cité, Paris 75006, France; Assistance Publique–Hôpitaux de Paris, Department of Digestive Oncology, Hôpital Européen Georges Pompidou, SIRIC CARPEM, Paris 75015, France

**Keywords:** fertility preservation, gastrointestinal cancer, oncofertility, young adult cancer patients, European oncology practices

## Abstract

**Introduction:**

The rising incidence of early-onset gastrointestinal (GI) cancer has made the impact of treatments on fertility of high significance. While there is abundant evidence on oncofertility outcomes in breast cancer and hematological malignancies, data regarding these perspectives in GI cancers is lacking. We sought to evaluate current practices of fertility preservation (FP) among GI oncologists across Europe.

**Methods:**

A cross-sectional survey was distributed through the Gastrointestinal Tract Cancer Group (GITCG) of the EORTC network and affiliated cooperative groups and cancer centers using a 10-item electronic survey regarding oncofertility practices. The target population was oncologists who routinely treat GI cancers. A statistical analysis was performed based on country, patient volume, and tumor type.

**Results:**

Two hundred and twenty-six GI oncologists from 27 countries completed the survey, the majority from high volume cancer centers. Fifty seven percent of the participating oncologists routinely discuss the impact of treatment on fertility in any patient <40 years, while 36% discuss this only in the curative setting. Fifty-nine percent refer female patients to standard FP options (embryo/oocyte preservation), while 24% chose to refer to ovarian cryopreservation. Of note, 17% indicated they would not refer a curative patient for FP at all due to time or resource issues. Sixty five percent routinely refer male patients to sperm preservation. Use of Gonadotropin Releasing Hormone analogues (GnRHa) in CRC patients is recommended by 34% of oncologists. In the setting of pelvic radiation, 65% refer a female patient for ovarian transposition before pelvic irradiation; 32% would consider uterine transposition. Sixty one percent would consider a nonradiation protocol as perioperative chemotherapy as a valid option for young female patients. We observed heterogeneity upon country but not upon physician gender.

**Conclusion:**

Our study indicates a substantial diversity in current practices in Europe with regard to FP in young cancer patients with GI malignancies, which is not always aligned with current guidelines. There is a need to disseminate and educate GI oncologists on oncofertility perspectives and contemporary data. Additionally, there is a need to establish evidence on the utility of fertility preservation options for patients with GI cancers.

Implications for Practice With early-onset gastrointestinal (GI) cancers on the rise, fertility preservation (FP) has become a critical aspect of supportive care in young patients. This pan-European survey reveals significant variation in routine discussion of FP and in the use of FP strategies, such as GnRH analogues and reproductive organ transposition. These findings highlight an urgent need for clinician education, clearer guidelines, and evidence-based strategies to ensure that all young GI cancer patients receive equitable, informed fertility care.

## Introduction

Recent data indicate an increase in the incidence of early-onset colorectal cancer (EOCRC), while approximately 13% of all colorectal cancers (CRC) are diagnosed in individuals under the age of 50.^1^^,^^2^ Furthermore, the steepest rise had been documented among young adults in the 20- to 30-year-old age group, thus, posing several clinical implications and challenges including long-term treatment-related toxicities, survivorship issues, and psychosocial unmet needs,^3^ as the combination of improved colorectal cancer survival rates and the trend towards delayed childbearing has made the issue of the impact of cancer treatments on fertility of high significance. While there is abundant evidence on reproductive outcomes of anticancer treatment protocols used in breast cancer patients and hematological malignancies, data regarding the reproductive outcomes of treatment protocols of CRC are lacking. The treatment for localized CRC typically involves chemotherapy, surgery and for locally advanced rectal cancer, also pelvic radiation. The potential negative impact of these treatment modalities on female reproductive system and sexual function are largely unknown. Studies performed in patients treated with 5FU for breast cancer demonstrated very mild gonadotoxic impact and generally no significant effect on fertility.^4^^,^^5^ Limited preclinical data confirmed this notion as well.^5^^,^^6^ However, the impact of oxaliplatin, which constitutes the backbone of chemotherapeutic regimens for CRC, on gonadal function remains largely unknown.

Standard fertility preservation methods include embryo or oocyte cryopreservation as well as oophoropexy for patients who are candidates for pelvic irradiation.^7^ Ovarian cryopreservation may serve as an ancillary method to preserve future fertility in case standard oocyte/embryo preservation is not feasible due to time constraints, as ovarian stimulation and oocyte retrieval requires at least 2 weeks. The use of Gonadotropin releasing Hormone analogs (GnRHa) has been implemented partially into clinical practice guidelines, mainly for breast cancer patients (ASCO guidelines^7^) due to lack of concrete evidence of clinical benefit. GnRHa had been evaluated in several clinical trials, all in breast cancer or lymphoma patients, indicating a relatively protective effect in breast cancer patients and a less definite impact in lymphoma patients.^8–13^ Nevertheless, there is currently no evidence regarding the efficacy of GnRHa in the setting of other cancer types in which non-cyclophosphamide-based protocols are employed.

Concerns regarding future fertility may greatly impact survivors of early-onset CRC, in a multifactorial manner encompassing psychological, sexual, and physical aspects.^14^ Fertility is a major issue for young cancer patients. Studies suggest that, particularly among young women, it is associated with emotional distress and poor quality of life (QoL).^15^ Those patients are also concerned about entering treatment-related early menopause.^16^ We aimed to assess current practice patterns of gastrointestinal (GI) oncologists from mid-large cancer centers across Europe concerning referral for fertility preservation (FP) and adherence to current clinical practice guidelines.

## Methods

### Fertility survey

A cross-sectional survey was distributed via the EORTC (European Organization for Research and Treatment of Cancer) and affiliated cooperative groups and cancer centers to collect data regarding FP practices among oncologists treating young adult patients with GI cancers. The survey was conducted electronically in January and February 2025. The questionnaire, created and administered through the Jotform online platform consisted of 10 items addressing institutional characteristics and clinical practices related to FP in young patients with colon, gastric or rectal cancer.

Questions covered information about the respondent’s medical center, including country and average annual number of young CRC patients seen (defined as patients aged <50 years). The survey focused on clinician practices concerning FP discussions with patients, including whether such conversations occur routinely, and how they may vary based on patient age and gender. Respondents were asked which FP options are typically offered at their institution, including both standard and nonstandard interventions.

Specific FP methods addressed in the survey included the use of GnRHa for ovarian suppression in female patients with colorectal and gastric cancers, as well as ovarian and uterine transposition for female rectal cancer patients anticipated to undergo pelvic radiation. Additionally, the survey assessed whether radiation-free treatment strategies are considered in female rectal cancer patients as in the PROSPECT study.^17^ The survey also queried barriers to initiating FP discussions, including institutional, logistical, and patient-related challenges. The full questionnaire is provided as Supplementary Figure 1.

### Data analysis

Comprehensive quantitative analysis of questionnaire data was performed using descriptive statistics and cross-tabulations. Categorical response distributions were explored by country, gender, and patient volume using normalized stacked bar charts. Centers were stratified based on quartiles of annual patient volume, enabling subgroup comparisons. Chi-square tests of independence were applied to evaluate differences in responses across gender and volume-based groups. Specific comparisons (eg, FP strategies) were refined to key categories for targeted analysis.

## Results

**Figure 1.**
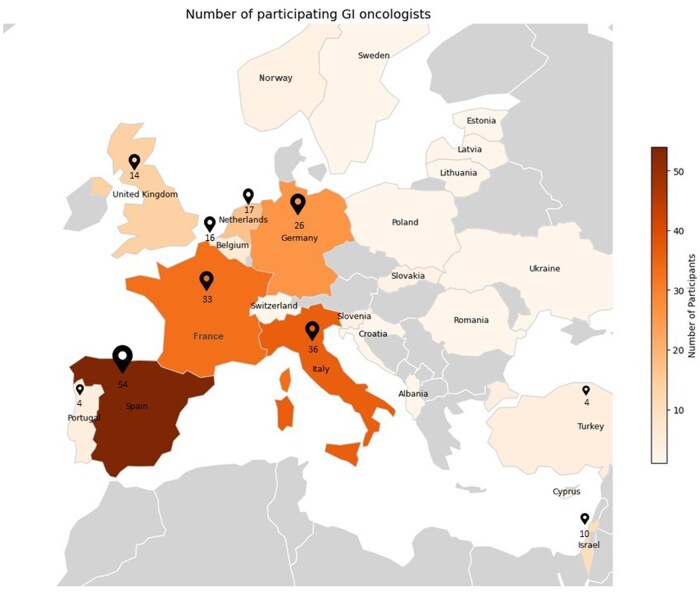
Geographic distribution of survey participants. The map illustrates responses from 226 gastrointestinal (GI) oncologists across 27 countries who completed the survey. Countries participating in the survey are shaded according to the number of responding oncologists, with a color gradient representing increasing participation. To preserve visual clarity, countries with three or fewer participants are not labeled with specific numerical values among them also Japan, India, and Canada that are not included in the map display.

A total of 226 oncologists from 27 countries completed the survey (Figure 1). Of the medical centers represented, 174 reported treating ≥10 young GI cancer patients (<50 y) annually and were classified as high-volume centers (Q2-Q4). Centers that reported treating fewer than 10 patients annually and were considered low-volume (*n *= 40). Fifty three percent of responders were female (*n* = 119) and no significant differences were observed in response patterns based on gender.

### Fertility preservation discussions

**Figure 2.**
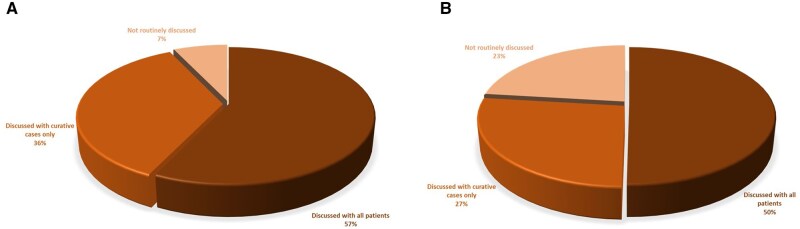
Routine discussion of the gonadal impact of anticancer treatments with female patients with GI cancer. (A) Among patients underthe age of 40 years, 57% of oncologists reported discussing fertility with all patients, while 36% limited the discussion to curative cases, and 7% did not routinely address the topic. (B) For patients under the age of 50 years, discussions with all patients dropped to 50%, with 27% limiting discussions to curative cases, and 23% not discussing fertility routinely.

Regarding discussing the potential impact of oncologic treatments on fertility with patients under the age of 40, 36% of the responders stated they conduct these discussions only with patients undergoing treatment with curative intent, and 57% would discuss with any young patient, including in the metastatic setting (Figure 2). The rates declined to 27% and 50%, respectively, when patient age was below 50 y. Notably, differences in response patterns were observed across European regions (Supplementary Figure 2).

### Fertility preservation methods

**Figure 3.**
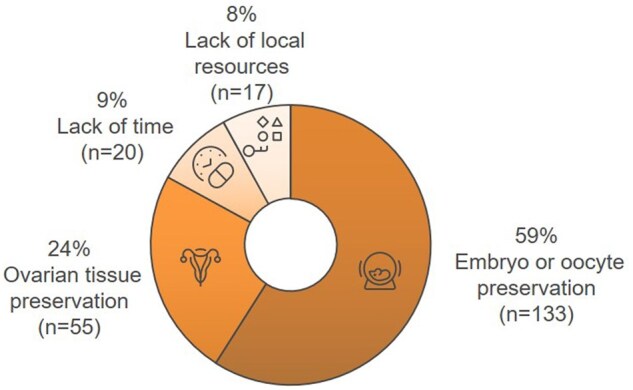
Referral of female patients with curative intent for fertility preservation methods before treatment. The majority of respondents (59%, *n* = 133) refer to embryo or oocyte cryopreservation, while 24% (*n* = 55) refer to ovarian tissue preservation. Referral is limited in some cases due to lack of time (9%, *n* = 20) or local resources (8%, *n* = 17).

**Figure 4.**
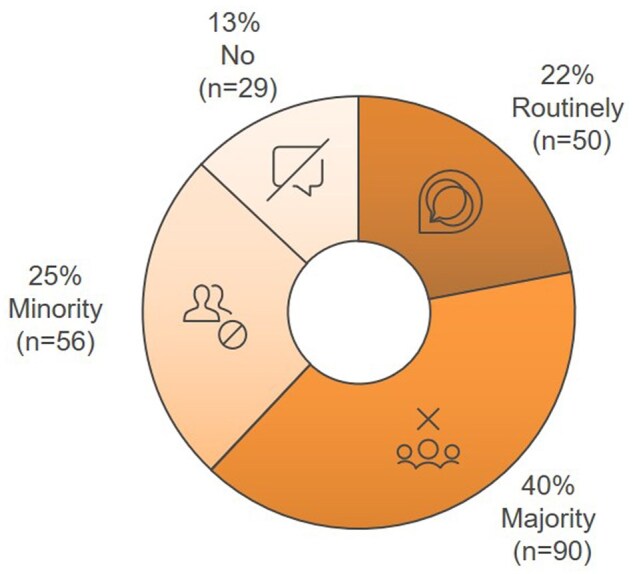
Referral of male patients for sperm preservation before chemotherapy. Sperm preservation is routinely recommended by 22% (*n* = 50) of oncologists; 40% (*n* = 90) refer the majority of eligible patients, 25% (*n* = 56) refer a minority, and 13% (*n* = 29) do not refer at all.

Participants referred to FP methods for female patients as follows: 59% of oncologists reported referring to oocyte or embryo cryopreservation; 24% reported referring patients for ovarian tissue cryopreservation. However, 8% indicated that FP was not offered due to a lack of institutional resources, and 9% cited treatment urgency as a barrier to referral (Figure 3). For male patients, 87% of oncologists reported referring them for sperm preservation in most instances but not routinely (Figure 4).

### Gonadal protection strategies

**Figure 5.**
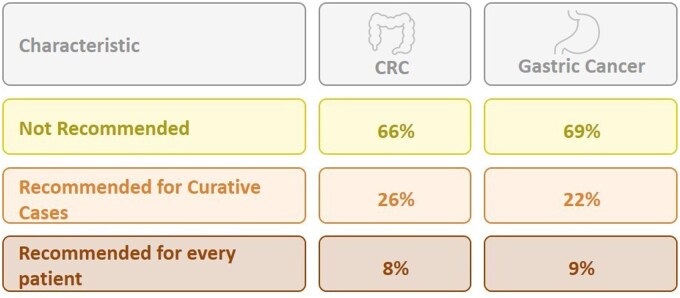
GnRHa prescription for female patients with CRC or gastric cancer before treatment initiation.

**Figure 6.**
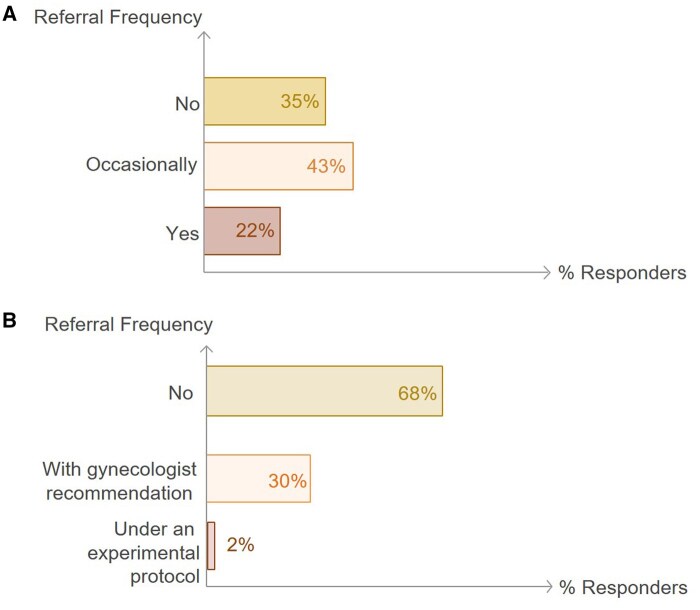
Reproductive organ transposition in female rectal cancer patients prior to radiation. Distribution of responses regarding ovarian transposition (A) and uterine transposition (B).

Use of GnRHa for ovarian suppression is routinely prescribed by 34% of respondents for patients with CRC and by 31% for patients with gastric cancer (Figure 5). The distribution of the answers to these questions differed significantly (*P* < .05) across countries. Among patients with rectal cancer scheduled to undergo radiation therapy, 67% of respondents reported referring patients for ovarian transposition; however, only 22% offered this option to all eligible patients (Figure 6A). In contrast, 32% of respondents noted that they would consider uterine transposition, based on recommendations from a gynecologist or within a research protocol (Figure 6B).

Sixty one percent of respondents indicated that they would consider offering a nonradiation treatment approach, as outlined in the PROSPECT study, for eligible patients with rectal cancer.

## Discussion

Fertility represents the main aspect affected by treatment-induced gonadal toxicity. Other hormonal-related domains are potentially altered due to gonadal function impairment, as neurocognitive, cardiovascular and psychosocial spheres (including body image and sexuality) also represent key issues for young cancer patients. The term “Oncofertility,” coined in 2006, founded a multidisciplinary interface between reproductive endocrinology and oncology with the goal to increase oncologists’ awareness to refer patients for FP and implementation of clinical practice guidelines.

We aimed to assess current practices of FP among GI oncologists across Europe to point at potential unmet needs. We employed the GITCG group of the EORTC network and affiliated cooperative groups and cancer centers using a 10-item electronic survey regarding oncofertility practices. Target population was oncologists who treat GI cancers routinely. Our results represent a diverse profile of 226 GI oncologists from 27 countries, the majority from high volume cancer centers, and indicate a suboptimal adherence to FP guidelines—only 57% of the participating oncologists discuss impact on fertility in any patient <40 years (36% discuss only in the curative setting). Of the cases who are referred for FP, 59% refer female patients to standard FP options (embryo/oocyte preservation), and 24% choose to refer to ovarian cryopreservation although this method is not considered as the golden standard. Interestingly, 17% indicated they would not refer a curative patient for FP at all due to time or resources issues. Sixty five percent would refer male patients to sperm preservation, though this procedure is significantly shorter (within 1-2 days) than FP in female patients.

It had been previously implicated by several studies that adherence to FP guidelines among oncologists generally remains suboptimal.^18–20^ A Canadian study that sought to assess clinicians’ perspectives on barriers to discussing fertility with young cancer patients indicated that clinicians’ unfamiliarity with infertility risks, FP technologies, referral processes, and procedures, as well as their perceptions on FP, influenced their practices regarding fertility discussions.^21^ The study implied that although clinicians acknowledged the importance of FP discussions, most reported feeling unprepared to discuss it with patients with cancer. A recent study conducted among young GI cancer patients in the US indicated that less than 50% of the patients with CRC reported that their healthcare team involved in their cancer care had discussed FP options prior to initiating anticancer treatment.^22^

While there is mounting evidence on oncofertility perspectives in the setting of breast cancer and hematological malignancies, there is paucity of data regarding these outcomes in young patients with GI cancer. CRC has been traditionally considered as a disease of older individuals, hence, reproductive outcomes were not an integral part of a comprehensive holistic treatment approach for the average patient. Due to the evolving phenomenon of early-onset CRC, GI oncologists have commonly been facing the treatment challenges of young patients, which dictates a dynamic paradigm shift in their supportive care. A summary of fertility preservation methods reported in GI cancer patients, along with available evidence for each approach, is presented in Supplementary Figure 3.

Upon our results, use of GnRHa in CRC patients is routinely recommended by 34% of oncologists. The role of GnRHa in preserving ovarian reserve and function has been constantly debated. While several studies indicated a positive effect in the setting of breast cancer,^8^^,^^9^ and others showed conflicting evidence in hematological malignancies,^10^ it has never been studied in CRC patients, and its clinical utility in the setting of oxaliplatin-based protocols remains to be elucidated. There are several possible mechanisms through which GnRHa may shield the ovary during chemotherapy such as reducing the levels of gonadotropins and hence placing the ovary in an artiﬁcial prepubertal state, resulting in inhibition of follicular recruitment in the ovaries and reduced primordial follicle burnout.^11^^,^^12^ Nevertheless, preclinical studies indicate that the effect of GnRHa on the ovary is not universal and depends upon the pattern of chemotherapy-induced gonadotoxicity, which differs upon treatment protocol. Most of the clinical studies evaluated the benefit of GnRHa in alleviating alkylating agent-induced ovarian toxicity, but these drugs are not a part of the treatment paradigm of CRC. Of note, the use of GnRHa carries substantial side effects which resemble menopausal symptoms such as hot ﬂushes, headaches, mood changes, sweating, and decreased bone density,^13^^,^^23^ and may enhance these symptoms that may also be due to chemotherapy-related ovarian dysfunction.

Fertility preservation ESMO guidelines set GnRHa in the general female FP flowchart, nevertheless, a deep dive into the discussion concludes that based on current evidence it should be considered as standard option for ovarian function preservation in premenopausal breast cancer patients undergoing systemic cytotoxic therapy, and may be discussed for other malignancies despite limited evidence, also in the context of its other potential effects, such as prevention of menometrorrhgia.^24^ In the recently published American Society of Clinical Oncology (ASCO) guidelines ovarian suppression by GnRHa is not considered a part of the established recommendations for cancer patients with the exception of breast cancer, due to lack of data in nonbreast cancer types.^25^

There is very limited evidence on the gonadal effect of oxaliplatin. A small retrospective study evaluated treatment-induced amenorrhea in premenopausal CRC patients who received FOLFOX treatment while of the 49 patients that were included in the analysis, 20 patients (41%) experienced amenorrhea during chemotherapy. Eight (16%) of those 20 had persistent amenorrhea 1 year after completion of chemotherapy. The overall incidence of amenorrhea during chemotherapy trended toward being higher in patients older than 40.^26^ In a translational study it has been demonstrated in a mouse model that oxaliplatin had a transient gonadotoxic effect on both gonads, while in a prospective evaluation of gonadal function in a small cohort of CRC patients treated with oxaliplatin-based protocols, reduced post-treatment AMH levels (a marker for ovarian reserve) and temporary amenorrhea were observed, though with a high rate of resumption of menses.^27^ This data implies that oxaliplatin may induce ovarian impairment, though differentially from chemotherapy-induced ovarian toxicity in breast cancer patients as formerly reported.^28^

Radiation-induced ovarian toxicity had been established, while ovarian transposition prior to radiation is considered a simple and effective procedure in preserving ovarian function, irrespective of the main goal of FP. In contrast, data regarding the effect of modern rectal irradiation protocols on uterine function is extremely limited, implying that pelvic irradiation in rectal cancer patients significantly affected uterine anatomy and perfusion observed by dynamic contrast-enhanced MRI.^29^ Our results demonstrate that in the setting of pelvic radiation, 65% refer a female patient for ovarian transposition before pelvic irradiation; 32% would consider uterine transposition.

Uterine transposition has been suggested in the setting of FP in patients who are candidates for pelvic irradiation. The procedure is not mentioned in the ESMO FP guidelines^24^ and is cited as an investigation approach in the recent ASCO guidelines.^25^ It should be noted that current evidence on uterine transposition is based upon case reports only,^30^ and therefore, should be taken carefully due to limited data regarding efficacy and safety.

Moreover, a radiation-free treatment strategy had been validated prospectively in the PROSPECT study^17^ and may therefore serve as an appealing option for young female patients who meet the inclusion criteria of the study. Indeed, 61% of our study participants stated they would consider a nonradiation protocol as perioperative chemotherapy as a valid option for young female patients.

The limitations of our study are in accordance with survey-based research, while our sample though broad may not represent all European GI oncologists. The survey was collected anonymously (without physician name, though hospital location was collected), and hence social bias was minimized.

In conclusion, our study reveals substantial heterogeneity in current practices across Europe concerning fertility preservation in young cancer patients with GI malignancies, which is not always aligned with current guidelines. The data attained in the study will serve as the basis to design future studies. There is a need for dissemination and education of oncofertility perspectives and contemporary data among GI oncologists as well as to establish evidence on the utility of FP options in the subset of GI cancer patients. Furthermore, the data obtained in our study shed light on the need to design and conduct prospective studies to evaluate the role of GnRHa in the setting of CRC protocols as well as to assess gonadal related psychosocial and neurocognitive perspectives that may lead to the implementation of interventions to improve supportive care in young GI cancer patients.

## Supplementary Material

oyaf350_Supplementary_Data

## Data Availability

Data generated by the study will be made available upon request.
