# Supplementary material for: Fertility preservation practices and gastrointestinal oncologist in Europe: a pan-European study
Source: Oncologist. 2025 Nov 5;30(11):oyaf350. doi: 10.1093/oncolo/oyaf350 (PMC12628310; doi:10.1093/oncolo/oyaf350)
Supplement: oyaf350_Supplementary_Data [file oyaf350_supplementary_data.zip › Supp figure 1 European Survey.pdf]

In order to submit this form, you should open it with Adobe Acrobat Reader.

EORTC
Gastrointestinal Tract Cancer Group

European Survey regarding fertility preservation in early-onset GI cancer

The combination of rising incidence of early-onset GI cancer and the trend towards delayed childbearing has made the impact of treatments on fertility of high significance. While there is abundant evidence on reproductive outcomes of anti-cancer treatment protocols used in breast cancer patients and hematological malignancies, data regarding the reproductive outcomes of treatment protocols of CRC are lacking. Gonadotropin releasing Hormone analogs (GnRHa) are commonly used for ovarian suppression in breast cancer patients or female patients with hematological malignancies. We sought to evaluate the implementation of fertility preservation measures in young patients with GI cancer, with an emphasis on the use of GnRHa in female patients.

Researcher details:

Researcher Name *

Site *

Country *

How many newly diagnosed patients with colon cancer and candidates for chemotherapy with curative intent who are below 50y are managed at your center per year?

Do you routinely discuss potential impact of anti-cancer treatment with every female patient with GI cancer below the age of 40 years?

- ○ Yes, for every patient
- ○ Yes, for patients with curative intent only
- ○ Usually not

Do you routinely discuss potential impact of anti-cancer treatment with every male patient with GI cancer below the age of 50 years?

- ◯ Yes, for every patient
- ◯ Yes, for patients with curative intent only
- ◯ Usually not

What type of fertility preservation measures do most female patients with curative intent at your institution undergo prior to chemotherapy:

- ◯ Embryo or oocyte preservation
- ◯ Ovarian tissue preservation
- ◯ Would not perform due to lack of time
- ◯ Would not perform due to lack of local resource

Do you refer male patients for sperm preservation?

- ◯ Yes, routinely
- ◯ Yes, in the majority of cases
- ◯ Yes, in the minority of cases
- ◯ Usually not

Do you (or the reproductive endocrinologist at your institution)routinely prescribe GnRHa for ovarian suppression for female patients with CRC or Gastric cancer?

	Yes, for every patient	Yes, for patients with curative intent only	No, I do not recommend GnRHa
CRC	◯	◯	◯
Gastric Cancer	◯	◯	◯

Do you routinely refer female patients with rectal cancer who are candidate for radiation to ovarian transposition?

- ◯ Yes, for every patient
- ◯ Occasionally
- ◯ No

Do you refer female patients with rectal cancer who are candidatefor radiation to uterine transposition?

- ◯ Yes, under an experimental protocol
- ◯ Sometimes, depends on the recommendation of the Gyncolleagues
- ◯ No

Do you consider a radiotherapy-free strategy as in PROSPECT study a preferable option for young female patients with rectal cancer (if resemble the inclusion criteria of the study)?

- ◯ Yes

◯ No

What barriers do you encounter when discussing fertility preservation options with patients?

◯ Lack of time for fertility preservation procedures before cancer treatment
◯ Limited local resources for fertility preservation
◯ Limited awareness or knowledge among oncology staff about fertility preservation options
◯ Emotional burden of discussing fertility preservation with patients
◯ Other ______
