# Supplementary figures and images for: Fertility preservation practices and gastrointestinal oncologist in Europe: a pan-European study
Source: Oncologist. 2025 Nov 5;30(11):oyaf350. doi: 10.1093/oncolo/oyaf350 (PMC12628310; doi:10.1093/oncolo/oyaf350)

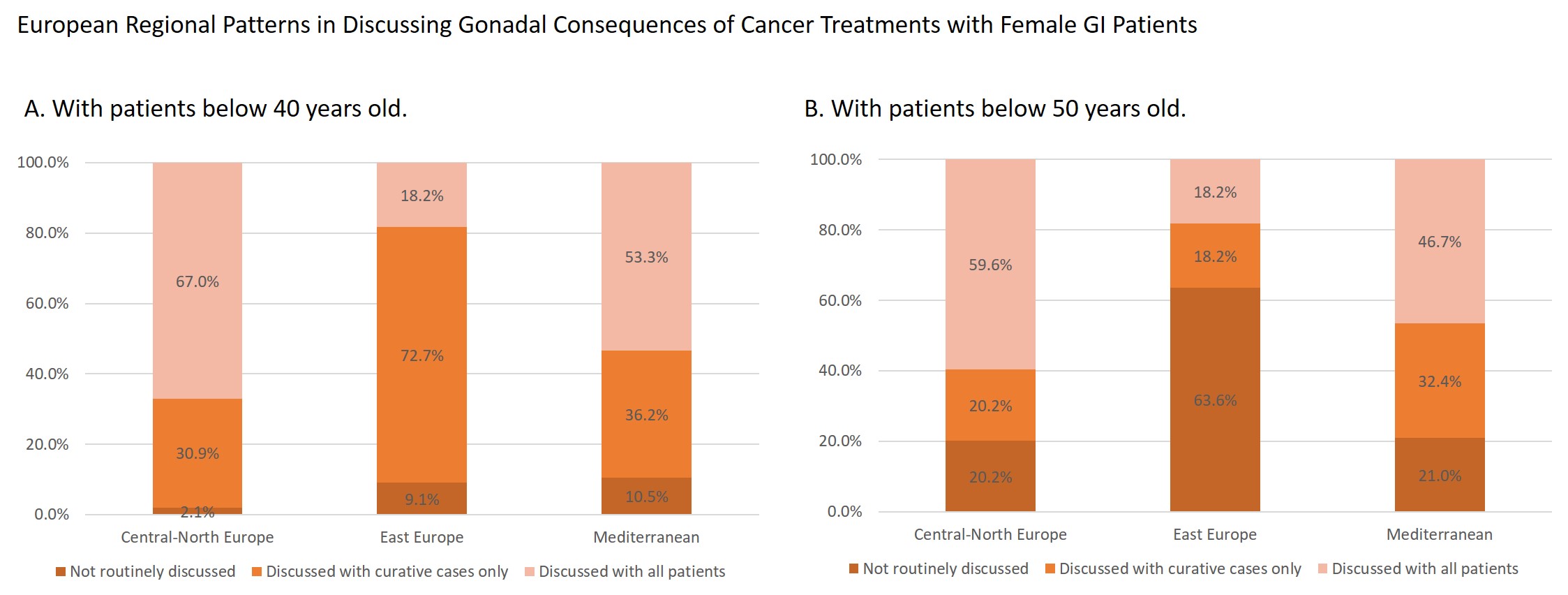

Supplement: oyaf350_Supplementary_Data [file oyaf350_supplementary_data.zip › Supp fig 2 regional distribution.jpg]

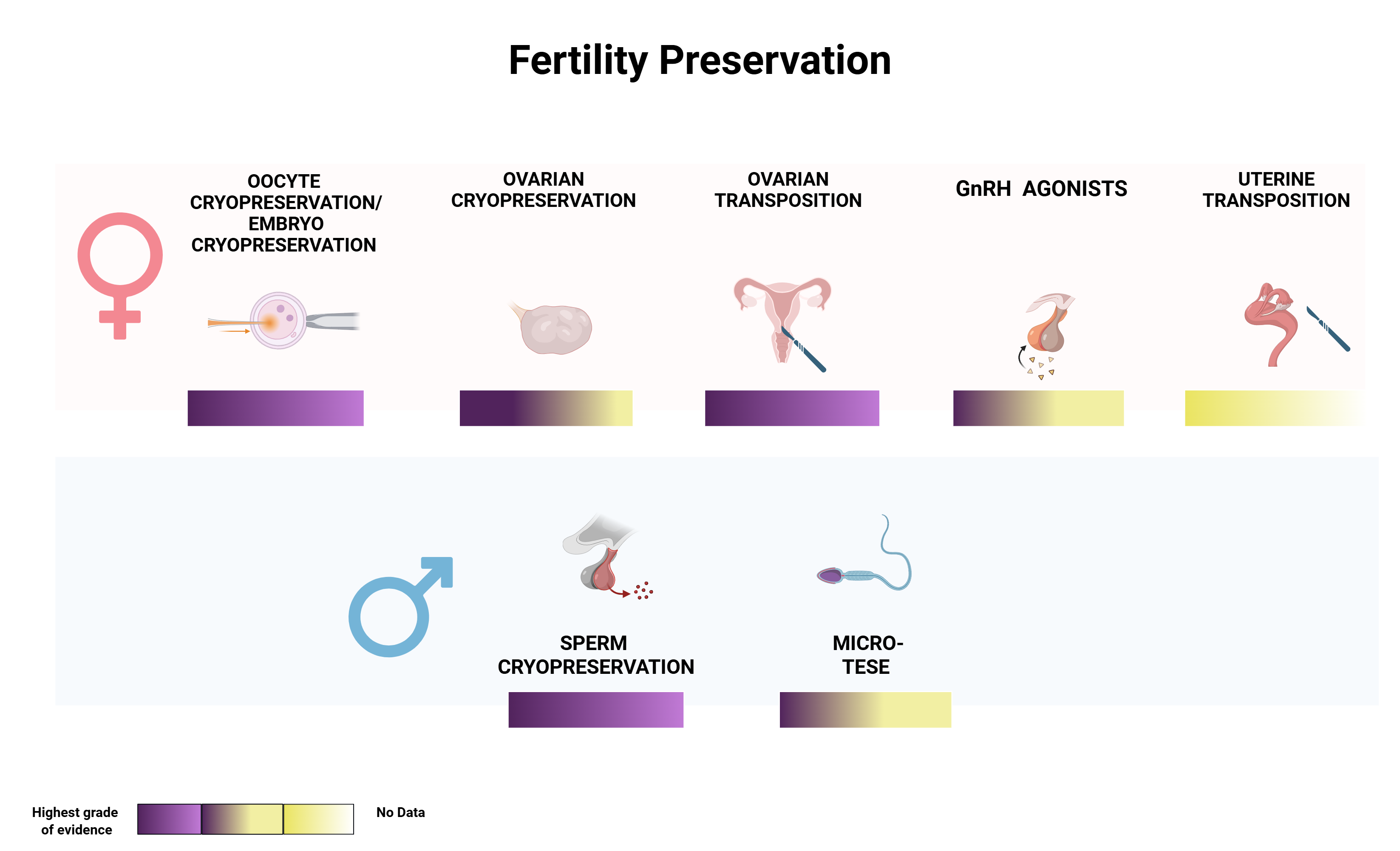

Supplement: oyaf350_Supplementary_Data [file oyaf350_supplementary_data.zip › Supp fig 3 Oncofertility in GI Cancers.png]
